# Molecular epidemiology of panton valentine leukocidin-producing *Staphylococcus aureus* infections, Djibouti, 2018–2023

**DOI:** 10.1371/journal.pntd.0013544

**Published:** 2025-09-30

**Authors:** Maxime Danjean, Virginie Courbin, Aurore Ho, Aurore Bousquet, Christophe Rodriguez, Hervé Jacquier, Paul-Louis Woerther

**Affiliations:** 1 Unité de Bactériologie, Département de Prévention, Diagnostic et Traitement des Infections, Hôpital Henri Mondor, APHP, Créteil, France; 2 UR 7380 DYNAMYC, Université Paris-Est Créteil, Créteil, France; 3 Département de Biologie, Hôpital Bégin, HNIA (Hôpital National d’Instruction des Armées), Saint-Mandé, France; 4 Plateforme de Génomique Genobiomics, Hôpital Henri Mondor, APHP, Créteil, France; 5 INSERM U955, Institut Mondor de Recherche Biomédicale, Créteil, France; Universidad Nacional Autonoma de Mexico, MEXICO

## Abstract

Between 2018 and 2023, a genomic study was conducted in a military camp in Djibouti to investigate the molecular epidemiology of Panton-Valentine Leukocidin-producing *Staphylococcus aureus*. Among 43 isolates, Sequence Type 152 was predominant (72%), mainly associated with spa types t355 and t4235. Core-genome Multi-Locus Sequence Typing revealed two concurrent transmission dynamics: localized inter-human outbreaks and repeated introductions from external sources. Comparative genomics with other African Sequence Type 152 isolates showed similar levels of diversity, suggesting a widespread continental dissemination. These findings highlight the importance of genomic surveillance to better understand and control the spread of this virulent lineage in Africa.

## Author summary

Panton-Valentine Leukocidin-producing *Staphylococcus aureus* is associated with community-acquired skin and soft tissue infections. While its global distribution has been well studied, data remain scarce for many African countries. Most existing studies have focused on methicillin-resistant strains, overlooking methicillin-susceptible forms, which are often more common in the community. We conducted a genomic study of isolates collected from skin infections in a French military camp hospital in Djibouti between 2018 and 2023. Overall, 98% of the isolates were methicillin-susceptible, and 72% belonged to the ST152 lineage. Whole-genome analysis revealed transmission clusters, as determined by genomic-match using core-genome Multi-Locus Sequence Typing. Our findings were consistent with both person-to-person spread within the camp — suggesting rapid local outbreaks — and longer-term patterns likely reflecting independent episodes linked to introductions from external sources or occasional importation events. By comparing these isolates with publicly available African genomes, we identified strong genetic relatedness, supporting the regional circulation of this clone. Our findings provide valuable molecular data from an underrepresented region and highlights the need for broader genomic surveillance across Africa.

## Background

Skin and soft tissue infections (SSTI) caused by *Staphylococcus aureus* are one of the main causes of community infections [1]. Among the responsible strains, those expressing the Panton-Valentine leukocidin (PVL) seem to be particularly involved [2,3]. Indeed, PVL-producing strains have been implicated in cases of recurrent SSTI episodes marked by rapidly progressing lesions and poor response to standard antibiotic treatment [3–5]. While most studies focus on PVL-producing *S. aureus* (PVL-SA) isolates responsible for community-acquired SSTI in Western countries [3], their epidemiology in Africa remains poorly documented [6]. However, in the few publications devoted to this topic, certain clones (Sequence Type [ST] 15, 8, 30 and 152) seem to predominate [6].

Here, we describe the local molecular epidemiology of PVL-SA strains in a military camp in Djibouti, using whole genome sequencing (WGS). Our results evidenced predominance of ST152 strains, highlighting both inter-human transmission in the camp, and a passive influx of ST152 strains from outside. Further WGS analysis showed that the ST152 PVL-SA from Djibouti were embedded among the strains from other origins, with equivalent levels of diversity, suggesting a continent-wide spread.

## Methods

Ethics Statement: This study does not involve any medical data. Therefore, approval from an Institutional Review Board was not required. All experimental procedures were conducted in accordance with the ethical principles outlined in the Declaration of Helsinki.

From 2018 to 2023, we conducted a prospective monocentric study investigating the molecular epidemiology of non-redundant PVL-SA from clinical samples of patients receiving medical care at the French army camp “Centre Médico-Chirurgical InterArmées (CMCIA) Dominique Mattei” in Djibouti. This camp is located in an arid, urban coastal area, near the Ampouli international airport and primarily serves French military personnel and their families (for >2-year deployments) living in the camp, as well as foreign military personnel, and defense civilians. The microbiological diagnosis (culture, identification and antimicrobial susceptibility testing) was performed in the laboratory located in the CMCIA. Then, all *S. aureus* isolates were sent to the Begin military hospital (Saint-Mandé, France) for PVL detection by PCR (RIDA GENE PVL, R-Biopharm). When positive, isolates were stored at -80°C and sent to the Genobiomics platform in Henri-Mondor Hospital (Créteil, France) for WGS (Illumina). Raw reads for this study have been deposited in the European Nucleotide Archive under accession number PRJEB81520 (See S1 Table). Genomes were *de novo* assembled using shovill v1.1.0 (https://github.com/tseemann/shovill). The assessment of completeness and contamination levels was conducted by employing the lineage_wf pipeline from CheckM v1.2.0 [7]. Antimicrobial resistance and virulence gene content was investigated using the Resfinder (v4.6.0) and VirulenceFinder (v2.0) databases, respectively, implemented in Abricate v1.0.1 (https://github.com/tseemann/abricate). Genotyping was performed by Multi-Locus Sequence Typing (MLST v2.23.0; https://github.com/tseemann/mlst) and *spa*-typing (spaTyper v0.3.3). A maximum-likelihood phylogeny with gamma distribution (FastTree v2.1.10) was constructed using a recombination-filtered (Gubbins v3.3.1) core-genome alignment (Snippy v4.6.0). To determine the relatedness of the main ST genomes, the allelic distances of the core-genome MLST genes (cgMLST) [8] were determined using the chewBBACA v3.3.2 software. The Hierarchical Clonal Complex with a 24-allele cut-off (hierCC24) through the MSTreeV2 hierarchical clustering algorithm (GrapeTree v1.5.0) was used to determine genomic similarity for evidence of cross-transmission [9]. Whole-genome analysis revealed transmission clusters, as determined by genomic-match using core-genome Multi-Locus Sequence Typing. The allelic diversity of the predominant PVL-SA ST was extensively compared to external genomes from the Pathogen.watch database, focusing on African isolates identified through detailed metadata.

## Results

During the study period, 43 patients were diagnosed with SSTI, the majority of whom were soldiers (n = 34/43; 79%). The corresponding PVL-SA isolates responsible for infections were included and the corresponding genomes were correctly assembled (see S2 Table). Only one strain was positive for the methicillin resistance encoding *mecA* gene (n = 1/43; 2%). The four main STs were ST152 (n = 31/43; 72%), ST15 (n = 3/43; 7%), ST93 (n = 2/43; 5%) and ST80 (n = 2/43; 5%). The remaining five strains (n = 5/43, 12%) belonged to ST1, ST6, ST8, ST1153 and ST4217 (See summary of background in S3 Table). Within the ST152 PVL-SA, the main *spa* types were t355 (n = 18/31; 58%), t4235 (n = 10/31; 33%), t1299 (n = 2/31; 6%) and t4346 (n = 1/31; 3%).

**Fig 1:**
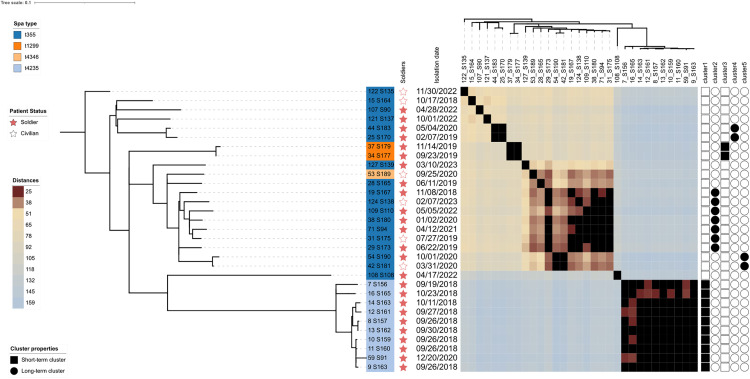
ST152 PVL-producing *S. aureus* phylogenetic tree and allelic distances from the Djibouti outbreak. The Djibouti ST152 *S. aureus* core-genome alignment-based phylogenetic tree is displayed on the left side of the figure, alongside the associated genomic metadata. The designation of the spa type is initially determined by the branch label, as indicated by the following colors: blue (t355), light blue (t4235), orange (t1299), and light orange (t4346). The isolation date of the bacterial strain is represented in the mm/dd/yyyy format. Stars labels represent patients status (red: soldier: white: civilian). The heatmap in the central panel illustrates cgMLST-based allelic distances, ranging from 1 to 159, represented by a color gradient from brown (25) to light blue (159). The black tiles represent distances below 24, which correspond to the hierCC24 affiliation. The short-period and the long-period hierCC24 assignments are represented by black squares and circles, respectively.

The median allelic distance among the ST152 genomes was 94 (range: [1; 159]) (Fig 1). Overall, 74% (n = 23/31) of the ST152 PVL-SA genomes grouped in five hierCC24 (described by genomes count - *spa* type - allelic distance range): cluster 1 (10 – t14235 – [1; 23]), cluster 2 (7 – t355 – [12; 24]), cluster 3 (2 – t1299 – 4), cluster 4 (2 – t355 – 11) and cluster 5 (2 – t355 – 6) (Fig 1 and S1 Fig). When comparing the date of sampling, we found that the clusters 1 and 3 (10 and 2 isolates, respectively) covered periods of 34 and 57 days, whereas the clusters 2, 4 and 5 (7, 2 and 2 isolates, respectively) covered periods of 4.2 years, 1.2 years and 6 months, probably reflecting different dynamic patterns of transmission. Note that in cluster 1, a late single episode (isolate 59_S91) was observed 2.2 years after the last isolate from the initial spread of the cluster, suggesting a hidden reservoir.

**Fig 2:**
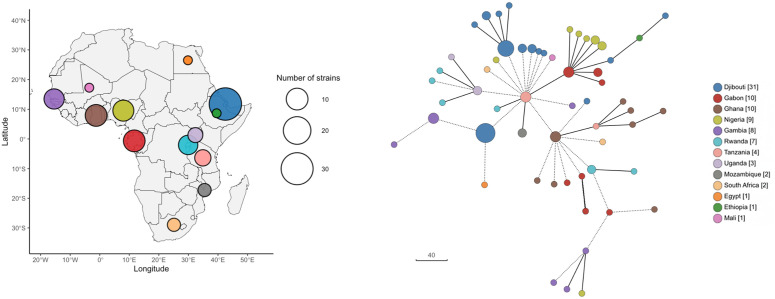
ST152 PVL-producing *S. aureus* molecular epidemiology in Africa. The left side of the figure illustrates the distribution of genomes across the African continent. The colored circles indicate the countries from which the genomes were derived for the purpose of genomic comparison by cgMLST. The size of the circles is proportional to the number of genomes. On the right side, the corresponding minimum spanning tree is plotted. Dotted lines represent distances exceeding 50 alleles, and genomes are collapsed when distances are below 24 to represent hierCC24. The size of the circles is correlated with the number of genomes within hierCC24. The country of origin is depicted with the same colors as the map on the left side. The map was created using the rnaturalearth v1.0.1 and rnaturalearthdata v1.0.0 R packages with an open-source, copyright-free Africa map provided by Natural Earth (http://www.naturalearthdata.com).

To better appreciate the diversity observed among ST152 strains in this study, we sought to compare our ST152 PVL-SA genomes with the African ones available from the Pathogen.watch database. Sixty-four genomes from 13 studies were found (See S4 Table). The comparison of the distributions of allelic distances of our strains and from the rest of Africa evidenced a similar magnitude (87; range [1; 142] *vs.* 87; range [1; 160], respectively). The hierarchical clustering analysis indicated a wide diversity in the core genome of available isolates circulating in Africa (Fig 2). These findings were consistent with the topology of the Minimum Spanning Tree linking genomes from distant countries, which suggest a diversity in the spread of ST152 PVL-SA across the African continent, including the strains from Djibouti.

In addition, the hierarchical clustering of all available genomes in the Pathogen.watch database and the present study was conducted. 62% of all ST152 PVL-SA were identified in Africa. However, the minimum spanning tree exhibited a heterogeneous distribution of ST152 PVL-SA, irrespective of their geographical distribution (see S2 Fig). Furthermore, the presence of genomes from multiple countries within the same HierCC24 category supports the hypothesis of international dissemination of this clone.

## Discussion

The WGS analysis of the PVL-SA responsible for infections in the Djibouti CMCIA “Dominique Mattei” military camp enabled us to depict a wide diversity of STs, dominated by the ST152. To gain a deeper understanding of the epidemiological pathways followed by this clone in the camp, cgMLST clustering was employed as a reproductive [10] and suitable tool for cross-transmission outbreaks investigation [9,11]. This approach enabled to detect various clusters spreading with different time lapses. While clusters 1 and 3 spread over short periods, suggesting rapid inter-human outbreaks inside the camp, clusters 2, 4 and 5 spread over longer periods, rather suggesting independent episodes linked with external reservoirs or sporadic influx from outside. Finally, we compared our ST152 PVL-SA with those available from the Pathogen.watch database to assess the diversity across the strains from Djibouti and those from other areas in Africa and worldwide. Interestingly, the strains from Djibouti were embedded among the strains from other origins, with equivalent levels of diversity, underlying the significant spread of ST152 PVL-SA across the continent. This was already suggested by previous observations highlighting the pathogenic role of the ST152 PVL-producing MSSA among asylum seekers from Eritrea as well as workers from South Africa [12,13]. Our study has some limitations, including the small number of isolates despite the long inclusion period, the absence of associated clinical data and the number of non-PVL-SA. For these reasons, the strength and generalizability of our molecular findings must be interpreted with caution. Further studies are required to assess the scale of epidemics caused by this clone to contain its spread.

## Supporting information

S1 Table Genomes of PVL-producing *S. aureus* available from Djibouti (BioProject PRJEB81520). (DOCX)

S2 Table Assembly quality metrics and cgMLST gene coverage of whole-genome sequenced PVL-SA isolates. (XLS)

S3 Table Genotyping and resistance genes of PVL-producing *S. aureus* strains isolated in Djibouti during 2018–2023. (DOCX)

S4 Table Genomes of PVL-SA available from the Pathogen.watch database. (DOCX)

S1 Fig Minimum spanning tree based on the allelic profiles of PVL-producing *S. aureus* from Djibouti. Dotted lines represent distances exceeding 50 alleles, and genomes are collapsed when distances are below 24 to represent hierCC24. The size of the circles is correlated with the number of genomes within hierCC24, and spa types are depicted with the same colors.
(TIF)

S2 Fig Worldwide minimum spanning tree based on the allelic profiles of ST152-PVL *S. aureus.* Dotted lines represent distances exceeding 50 alleles, and genomes are collapsed when distances are below 24 to represent hierCC24. The size of the circles is correlated with the number of genomes within hierCC24, and country origin are depicted by various colors.
(TIF)
