# Supplementary material for: Molecular epidemiology of panton valentine leukocidin-producing Staphylococcus aureus infections, Djibouti, 2018–2023
Source: PLoS Negl Trop Dis. 2025 Sep 30;19(9):e0013544. doi: 10.1371/journal.pntd.0013544 (PMC12483272; doi:10.1371/journal.pntd.0013544)
Supplement: S1 Table — (DOCX) [file pntd.0013544.s001.docx]

**Supplementary Table 1.** Genomes of PVL-producing *S. aureus* available from Djibouti (BioProject PRJEB81520)

| **Sample accession** | **Study id** | **Sample alias** | **Collection date (dd/mm/yyyy)** |
| --- | --- | --- | --- |
| SAMEA116330758 | 7_S156 | ERS21379604 | 19/09/2018 |
| SAMEA116330759 | 8_S157 | ERS21379605 | 26/09/2018 |
| SAMEA116330760 | 9_S163 | ERS21379606 | 26/09/2018 |
| SAMEA116330761 | 10_S159 | ERS21379607 | 26/09/2018 |
| SAMEA116330762 | 11_S160 | ERS21379608 | 26/09/2018 |
| SAMEA116330763 | 12_S161 | ERS21379609 | 27/09/2018 |
| SAMEA116330764 | 13_S162 | ERS21379610 | 30/09/2018 |
| SAMEA116330765 | 14_S163 | ERS21379611 | 11/10/2018 |
| SAMEA116330766 | 15_S164 | ERS21379612 | 17/10/2018 |
| SAMEA116330767 | 16_S165 | ERS21379613 | 23/10/2018 |
| SAMEA116330768 | 17_S166 | ERS21379614 | 02/11/2018 |
| SAMEA116330769 | 19_S167 | ERS21379615 | 08/11/2018 |
| SAMEA116330770 | 20_S168 | ERS21379616 | 12/11/2018 |
| SAMEA116330771 | 24_S164 | ERS21379617 | 30/01/2019 |
| SAMEA116330772 | 25_S170 | ERS21379618 | 07/02/2019 |
| SAMEA116330773 | 27_S171 | ERS21379619 | 13/03/2019 |
| SAMEA116330774 | 28_S165 | ERS21379620 | 11/06/2019 |
| SAMEA116330775 | 29_S173 | ERS21379621 | 22/06/2019 |
| SAMEA116330776 | 31_S175 | ERS21379622 | 27/07/2019 |
| SAMEA116330777 | 32_S176 | ERS21379623 | 20/08/2019 |
| SAMEA116330778 | 34_S177 | ERS21379624 | 23/09/2019 |
| SAMEA116330779 | 35_S178 | ERS21379625 | 03/10/2019 |
| SAMEA116330780 | 37_S179 | ERS21379626 | 14/11/2019 |
| SAMEA116330781 | 38_S180 | ERS21379627 | 02/01/2020 |
| SAMEA116330782 | 42_S181 | ERS21379628 | 31/03/2020 |
| SAMEA116330783 | 43_S182 | ERS21379629 | 09/04/2020 |
| SAMEA116330784 | 44_S183 | ERS21379630 | 04/05/2020 |
| SAMEA116330785 | 45_S184 | ERS21379631 | 08/01/2020 |
| SAMEA116330786 | 53_S189 | ERS21379632 | 25/09/2020 |
| SAMEA116330787 | 54_S190 | ERS21379633 | 01/10/2020 |
| SAMEA116330788 | 59_S91 | ERS21379634 | 20/12/2020 |
| SAMEA116330789 | 65_S92 | ERS21379635 | 07/03/2021 |
| SAMEA116330790 | 71_S94 | ERS21379636 | 12/04/2021 |
| SAMEA116330791 | 107_S90 | ERS21379637 | 28/04/2022 |
| SAMEA116330792 | 108_S108 | ERS21379638 | 17/04/2022 |
| SAMEA116330793 | 109_S110 | ERS21379639 | 05/05/2022 |
| SAMEA116330794 | 111_S110 | ERS21379640 | 04/04/2022 |
| SAMEA116330795 | 121_S137 | ERS21379641 | 01/10/2022 |
| SAMEA116330796 | 122_S135 | ERS21379642 | 30/11/2022 |
| SAMEA116330797 | 124_S138 | ERS21379643 | 07/02/2023 |
| SAMEA116330798 | 125_S134 | ERS21379644 | 25/02/2023 |
| SAMEA116330799 | 126_S133 | ERS21379645 | 03/03/2023 |
| SAMEA116330800 | 127_S139 | ERS21379646 | 10/03/2023 |
