# Supplementary material for: Molecular epidemiology of panton valentine leukocidin-producing Staphylococcus aureus infections, Djibouti, 2018–2023
Source: PLoS Negl Trop Dis. 2025 Sep 30;19(9):e0013544. doi: 10.1371/journal.pntd.0013544 (PMC12483272; doi:10.1371/journal.pntd.0013544)
Supplement: S3 Table — (DOCX) [file pntd.0013544.s003.docx]

**Supplemental Table 3.** Genotyping and resistance genes of PVL-producing *S. aureus* strains isolated in Djibouti during 2018-2023.

|  |  |  |  |
| --- | --- | --- | --- |
| **Genome** | **ST** | **spa type** | **Resistance genes** |
| **45_S184** | 1 | t127 | *blaZ, tetKLM, aph3, fusC* |
|  |  |  |  |
| **24_S164** | 6 | t304 | *blaZ* |
|  |  |  |  |
| **111_S110** | 8 | t008 | *blaZ, mecA* |
|  |  |  |  |
| **17_S166** | 15 | t085 | *blaZ, dfrG, tetKLM* |
| **20_S168** |  | t085 | *blaZ, dfrG* |
| **27_S171** |  | t085 | *dfrG* |
|  |  |  |  |
| **125_S134** | 80 | t1849 | *blaZ* |
| **65_S92** |  | t1849 | *blaZ* |
|  |  |  |  |
| **35_S178** | 93 | t202 | *blaZ, lnuA* |
| **43_S182** |  | t202 | *blaZ* |
|  |  |  |  |
| **107_S90** | 152 | t355 | *blaZ, dfrG* |
| **108_S108** |  |  | *blaZ* |
| **109_S110** |  |  | *blaZ, dfrG, fusC* |
| **121_S137** |  |  | *blaZ, dfrG, tetKLM* |
| **122_S135** |  |  | *dfrG* |
| **124_S138** |  |  | *blaZ, dfrG* |
| **127_S139** |  |  | *blaZ* |
| **15_S164** |  |  | *blaZ, dfrG* |
| **19_S167** |  |  | *blaZ, dfrG* |
| **25_S170** |  |  | *blaZ, dfrG, tetKLM* |
| **28_S165** |  |  | *blaZ, dfrG* |
| **29_S173** |  |  | *blaZ, dfrG* |
| **31_S175** |  |  | *blaZ, dfrG* |
| **38_S180** |  |  | *dfrG* |
| **42_S181** |  |  | *blaZ, dfrG* |
| **44_S183** |  |  | *blaZ, dfrG* |
| **54_S190** |  |  | *blaZ, dfrG* |
| **71_S94** |  |  | *blaZ, dfrG* |
| **34_S177** |  | t1299 | *blaZ, dfrG, tetKLM* |
| **37_S179** |  |  | *dfrG* |
| **10_S159** |  | t4235 | *blaZ, dfrG, ermC* |
| **11_S160** |  |  | *blaZ, dfrG, ermC* |
| **12_S161** |  |  | *blaZ, dfrG* |
| **13_S162** |  |  | *dfrG, ermC* |
| **14_S163** |  |  | *blaZ, dfrG, ermC* |
| **16_S165** |  |  | *blaZ, dfrG* |
| **59_S91** |  |  | *blaZ, dfrG, ermC* |
| **7_S156** |  |  | *dfrG* |
| **8_S157** |  |  | *blaZ, dfrG, ermC* |
| **9_S163** |  |  | *blaZ, dfrG, ermC* |
| **53_S189** |  | t4346 | *dfrG* |
|  |  |  |  |
| **32_S176** | 1153 | t903 | *none* |
|  |  |  |  |
| **126_S133** | 4271 | t355 | *blaZ, dfrG* |
|  |  |  |  |
|  |  |  |  |
