# Supplementary material for: Molecular epidemiology of panton valentine leukocidin-producing Staphylococcus aureus infections, Djibouti, 2018–2023
Source: PLoS Negl Trop Dis. 2025 Sep 30;19(9):e0013544. doi: 10.1371/journal.pntd.0013544 (PMC12483272; doi:10.1371/journal.pntd.0013544)
Supplement: S4 Table — (DOCX) [file pntd.0013544.s004.docx]

**Supplementary Table 4.** Genomes of PVL-producing *S. aureus* available from the Pathogen.watch database

| **Sample accession** | **Country** | **Collection date (dd/mm/yyyy)** | **Study accession** |
| --- | --- | --- | --- |
| SAMEA3432862 | Mali | unknown | PRJEB9499 |
| SAMEA3109319 | Tanzania | 01/01/2014 | PRJEB2655 |
| SAMEA3109324 | Tanzania | 01/01/2014 | PRJEB2655 |
| SAMEA3671740 | Tanzania | 01/01/2012 | PRJEB11627 |
| SAMEA3671744 | Tanzania | 01/01/2012 | PRJEB11627 |
| SAMEA3671753 | Gabon | 01/01/2011 | PRJEB11627 |
| SAMEA3671755 | Gabon | 01/01/2011 | PRJEB11627 |
| SAMEA3671756 | Gabon | unknown | PRJEB11627 |
| SAMEA3671757 | Gabon | unknown | PRJEB11627 |
| SAMEA3671765 | Gabon | 01/01/2011 | PRJEB11627 |
| SAMEA3671767 | Gabon | 01/01/2012 | PRJEB11627 |
| SAMEA3671768 | Gabon | 01/01/2012 | PRJEB11627 |
| SAMEA3671770 | Gabon | 01/01/2012 | PRJEB11627 |
| SAMEA3671771 | Gabon | 01/01/2012 | PRJEB11627 |
| SAMEA3671778 | Gabon | 01/01/2013 | PRJEB11627 |
| SAMEA3671785 | Mozambique | 01/01/2012 | PRJEB11627 |
| SAMEA3671787 | Mozambique | 01/01/2012 | PRJEB11627 |
| SAMEA3727062 | Gambia | 12/09/2005 | PRJEB12419 |
| SAMEA3727074 | Gambia | 25/09/2007 | PRJEB12419 |
| SAMEA3727077 | Gambia | 24/04/2008 | PRJEB12419 |
| SAMEA3727082 | Gambia | 10/02/2009 | PRJEB12419 |
| SAMEA3727087 | Gambia | 24/08/2009 | PRJEB12419 |
| SAMEA5330268 | Gambia | unknown | PRJEB31151 |
| SAMEA5330269 | Gambia | unknown | PRJEB31151 |
| SAMEA5330270 | Gambia | unknown | PRJEB31151 |
| SAMEA7375599 | Reunion | 01/01/2011 | PRJEB40651 |
| SAMEA7375611 | Reunion | 01/01/2014 | PRJEB40651 |
| SAMEA7375629 | Reunion | 01/01/2017 | PRJEB40651 |
| SAMEA7375659 | Mayotte | 01/01/2019 | PRJEB40651 |
| SAMEA7375662 | Mayotte | 01/01/2019 | PRJEB40651 |
| SAMEA7375671 | Mayotte | 01/01/2019 | PRJEB40651 |
| SAMEA7458207 | Uganda | 01/01/2018 | PRJEB40863 |
| SAMEA7458208 | Uganda | 01/01/2018 | PRJEB40863 |
| SAMEA7458226 | Uganda | 01/01/2018 | PRJEB40863 |
| SAMEA7471333 | Ethiopia | 01/05/2016 | PRJEB40908 |
| SAMEA7471339 | Nigeria | 01/05/2016 | PRJEB40908 |
| SAMEA7471340 | Nigeria | 01/05/2016 | PRJEB40908 |
| SAMEA7471341 | Nigeria | 01/05/2016 | PRJEB40908 |
| SAMEA7471348 | Nigeria | 01/05/2016 | PRJEB40908 |
| SAMEA7471349 | Nigeria | 01/05/2016 | PRJEB40908 |
| SAMEA7471354 | Nigeria | 01/05/2016 | PRJEB40908 |
| SAMEA7471355 | Nigeria | 01/05/2016 | PRJEB40908 |
| SAMEA7471357 | Nigeria | 01/05/2016 | PRJEB40908 |
| SAMEA7471359 | Nigeria | 01/05/2016 | PRJEB40908 |
| SAMEA7471415 | South Africa | 01/05/2016 | PRJEB40908 |
| SAMEA7471417 | South Africa | 01/05/2016 | PRJEB40908 |
| SAMN03658603 | Ghana | 03/04/2013 | PRJNA283747 |
| SAMN03658605 | Ghana | 09/01/2013 | PRJNA283747 |
| SAMN03658606 | Ghana | 03/04/2013 | PRJNA283747 |
| SAMN03658607 | Ghana | 09/01/2013 | PRJNA283747 |
| SAMN03658608 | Ghana | 03/04/2013 | PRJNA283747 |
| SAMN13616289 | Rwanda | 27/07/2018 | PRJNA596257 |
| SAMN15640965 | Egypt | 01/01/2015 | PRJNA648411 |
| SAMN16521182 | Ghana | unknown | PRJNA670821 |
| SAMN16521199 | Ghana | unknown | PRJNA670821 |
| SAMN16521200 | Ghana | unknown | PRJNA670821 |
| SAMN16521202 | Ghana | unknown | PRJNA670821 |
| SAMN16521203 | Ghana | unknown | PRJNA670821 |
| SAMN21888049 | Rwanda | 01/01/2016 | PRJNA767102 |
| SAMN21888054 | Rwanda | 01/01/2017 | PRJNA767102 |
| SAMN21888055 | Rwanda | 01/01/2017 | PRJNA767102 |
| SAMN21888066 | Rwanda | 01/01/2017 | PRJNA767102 |
| SAMN21888067 | Rwanda | 01/01/2017 | PRJNA767102 |
| SAMN21888071 | Rwanda | 01/01/2017 | PRJNA767102 |
