# Supplementary figures and images for: Molecular epidemiology of panton valentine leukocidin-producing Staphylococcus aureus infections, Djibouti, 2018–2023
Source: PLoS Negl Trop Dis. 2025 Sep 30;19(9):e0013544. doi: 10.1371/journal.pntd.0013544 (PMC12483272; doi:10.1371/journal.pntd.0013544)

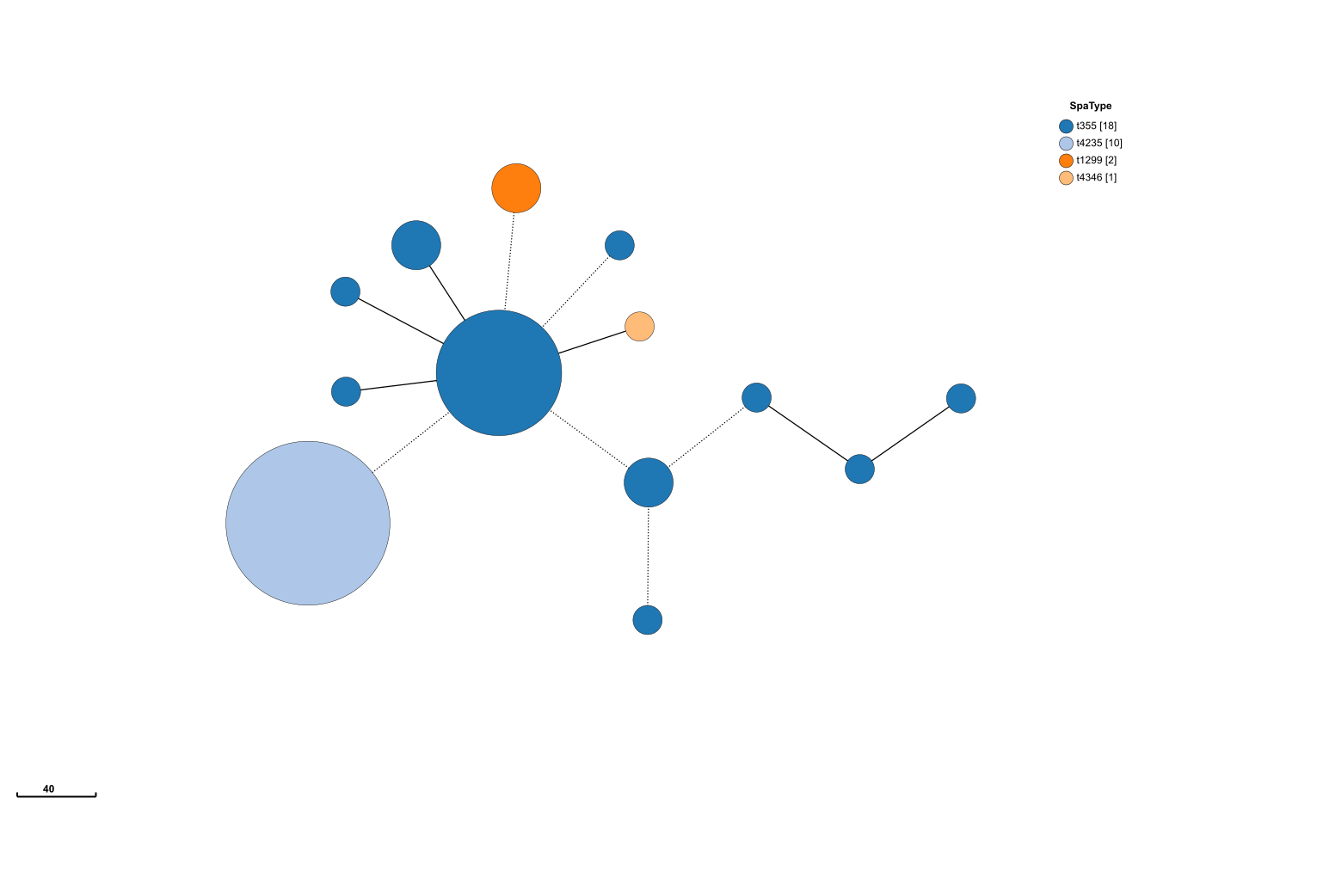

Supplement: S1 Fig — Dotted lines represent distances exceeding 50 alleles, and genomes are collapsed when distances are below 24 to represent hierCC24. The size of the circles is correlated with the number of genomes within hierCC24, and spa types are depicted with the same colors. (TIF) [file pntd.0013544.s005.tif]

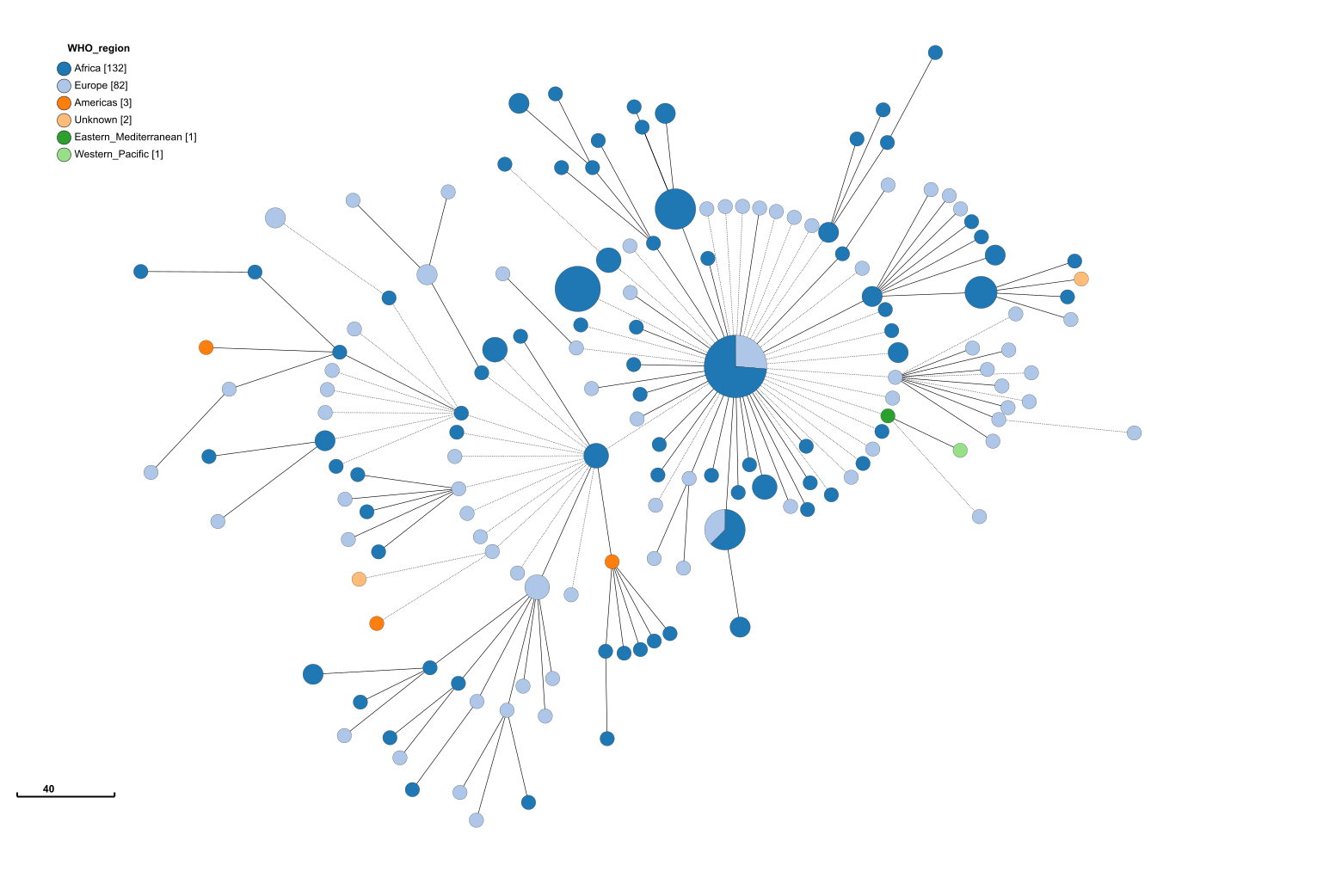

Supplement: S2 Fig — Dotted lines represent distances exceeding 50 alleles, and genomes are collapsed when distances are below 24 to represent hierCC24. The size of the circles is correlated with the number of genomes within hierCC24, and country origin are depicted by various colors. (TIF) [file pntd.0013544.s006.tif]
